# High Rosmarinic Acid Content *Melissa officinalis* L. Phytocomplex Modulates Microglia Neuroinflammation Induced by High Glucose

**DOI:** 10.3390/antiox14020161

**Published:** 2025-01-29

**Authors:** Giacomina Videtta, Chiara Sasia, Nicoletta Galeotti

**Affiliations:** Department of Neurosciences, Psychology, Drug Research and Child Health (Neurofarba), Section of Pharmacology and Toxicology, University of Florence, Viale G. Pieraccini 6, 50139 Florence, Italy; giacomina.videtta@unifi.it (G.V.); chiara.sasia@unifi.it (C.S.)

**Keywords:** neuroinflammation, microglia, diabetes, *Melissa officinalis* L., rosmarinic acid

## Abstract

Diabetic patients experience hyperglycemia, which can affect multiple organs, including brain function, leading to disabling neurological complications. Hyperglycemia plays a key role in promoting neuroinflammation, the most common complication in diabetic individuals, through the activation of microglia. Attenuating hyperglycemia-related neuroinflammation in microglia may reduce diabetes-associated neurological comorbidities. Natural remedies containing phenolic compounds have shown efficacy in mitigating microglia-mediated neuroinflammation. The aim of this study was to investigate the potential of a *Melissa officinalis* L. (MO) phytocomplex, obtained from plant cell cultures and enriched in its main polyphenolic constituent, rosmarinic acid (RA), in attenuating hyperglycemia-induced neuroinflammation in microglia. A time-course morphological analysis of BV2 microglial cells exposed to high glucose (HG) levels showed a shift towards a proinflammatory phenotype, peaking after 48 h, which was reversed by pretreatment with MO. Biochemical assays revealed increased expression of the microglial marker CD11b (187%), activation of the NF-κB pathway (179%), expression of iNOS (225%), enhanced phosphorylation of ERK1/2 (180%), and increased expression of the proinflammatory cytokine IL-6 (173%). Pretreatment with MO prevented the aberrant expression of these proinflammatory mediators and restored SIRT1 levels. Exposure of neuronal SH-SY5Y cells to the conditioned medium from HG-exposed microglia significantly reduced cell viability. MO counteracted this effect, exhibiting neuroprotective activity. RA showed efficacy comparable to that of MO. In conclusion, MO and RA attenuated microglia-mediated oxidative imbalance and neuroinflammation under HG exposure by inhibiting the morphological shift toward a proinflammatory phenotype induced by HG and abrogating the subsequent activation of the downstream ERK1/2–NF-κB–iNOS pathway.

## 1. Introduction

Diabetes is a metabolic disease characterized by hyperglycemia, which results from either defects in insulin secretion (type 1 diabetes) or insulin resistance, where insulin is produced but cannot effectively interact with its receptors (type 2 diabetes). According to the International Diabetes Federation, the number of adults with diabetes reached 463 million in 2019. The prevalence of diabetes is expected to rise to 600 million by 2030 and surge to 700 million by 2045 [1]. This rapid increase has been primarily linked to the rise in type 2 diabetes, a chronic, age-related disease that mainly affects middle-aged and older adults. The extended life expectancy in developed countries is contributing to a growing proportion of elderly individuals in the population, which, in turn, is increasing the number of people living with diabetes and its associated complications [2]. Additionally, an estimated 318 million adults have prediabetes or impaired glucose tolerance, placing them at a higher risk of developing diabetes [1].

Hyperglycemia can damage multiple organs, including the brain. In fact, diabetes is associated with several disabling neurological complications, such as peripheral neuropathy, diabetic retinopathy, diabetic nephropathy, and cognitive decline [3,4]. Among these, diabetes-related cognitive deficits and dementia are considered more severe than cardiovascular complications, making them the second leading cause of death in diabetic patients [5]. Furthermore, type 2 diabetes can increase the risk of Alzheimer’s disease by 1.5–2.5 times [6].

Although the precise mechanism underlying the link between diabetes and neurological disturbances has not been fully elucidated, several multifactorial pathways have been implicated, including oxidative stress, neuroinflammation, and cerebrovascular damage [7,8,9]. Neuroinflammation, an inflammatory response within the central nervous system primarily sustained by microglial cells, is the most prevalent complication in diabetic individuals [10] and a common factor in nearly all neurological disorders. Studies have shown that hyperglycemia is a significant factor that exacerbates oxidative stress and promotes the release of cytokines [11]. Indeed, hyperglycemia activates microglia and resident immune cells in the brain, playing a key pathological role in the promotion of neuroinflammation [12,13]. Therefore, the continuous hyperglycemic insult in diabetic patients exacerbates the release of proinflammatory mediators and promotes cellular morphological changes, sustaining a chronic inflammatory state that creates an unfavorable environment for neurons, potentially leading to neurodegeneration [14].

Numerous clinical trials have established that maintaining near-normal blood glucose levels mitigates the progression of complications associated with diabetes [15,16]. However, these approved synthetic drugs cannot always reverse the progression of comorbidities [17], and long-term use may be limited due to side effects or high costs, highlighting the urgent need for alternative therapeutics with fewer associated drawbacks. Although the precise mechanism linking neurological deficits and diabetes remains incompletely understood, the crucial role of neuroinflammation induced by activated microglial cells in diabetes-associated neurological comorbidities suggests that preventing excessive microglial activation could be a promising approach for early intervention.

Natural products are gaining recognition as therapeutic agents due to their lower cytotoxicity and reduced side effects compared to synthetic drugs [18]. Natural phenolic compounds have been shown to effectively mitigate microglia-mediated neuroinflammation [19], and some natural products have demonstrated efficacy in improving diabetes and its complications, as well as in reducing inflammatory responses [20]. Among these, *Melissa officinalis* L. (lemon balm) stands out as a particularly promising intervention. This medicinal plant has long been known for its anxiolytic and sedative properties, and recent evidence suggests its efficacy in alleviating depression and anxiety in diabetic patients [21]. *M. officinalis* also shows a safe and beneficial impact on lipid profiles and glycemic control in diabetic patients [22]. These effects are likely attributed to its main constituent, rosmarinic acid (RA), which has demonstrated potential antidiabetic activity in preclinical studies [23]. RA possesses both antioxidant and anti-inflammatory properties [24], making it particularly promising for attenuating microglia-mediated neuroinflammation in hyperglycemic conditions. Based on these considerations, we aimed to evaluate the efficacy of a *Melissa officinalis* L. (MO) phytocomplex enriched in rosmarinic acid (RA) in attenuating hyperglycemia-induced neuroinflammation. To better define the activity profile of MO, we also investigated the role of RA in MO-induced effects. The study employed an in vitro model of neuroinflammation induced by prolonged exposure of BV2 microglial cells to high glucose levels. Despite RA’s high therapeutic potential, its poor water solubility and low bioavailability limit its clinical application [25]. Additionally, the RA content in *Melissa officinalis* L. extract is highly variable [26], making it difficult to obtain preparations with a consistent and reproducible content of active constituents. To address these challenges, our study evaluated the efficacy of an *M. officinalis* L. phytocomplex obtained through in vitro plant cell cultures. This technique allows for the production of preparations with higher RA content, reduces the significant variability in phytoconstituent levels from crop-derived *M. officinalis*, enables the creation of reliable, standardized preparations, and offers a method for obtaining contaminant-free, standardized plant preparations in industrial quantities.

## 2. Materials and Methods

### 2.1. Cell Culture

Murine immortalized microglial BV2 cells (mouse, C57BL/6, brain, microglial cells (Tema Ricerca, Genova, Italy); 10–20 passages) were used. BV-2 were thawed and then cultured in a 75 cm^2^ flask with RPMI-1640 medium (Sarstedt, Milan, Italy) added with 10% of heat-inactivated (56 °C, 30 min) fetal bovine serum (FBS, Gibco, Milan, Italy), 1% of L-glutamine, and 1% penicillin-streptomycin solution (Merck, Darmstadt, Germany). Cells were cultured at 37 °C and 5% CO_2_ with every two-day media change until 70–80% confluence was achieved. Then, EDTA-trypsin solution (Sigma-Aldrich, Milan, Italy) was used to detach BV-2 from the flask, and cell counting was performed using Trypan blue staining. BV-2 cells were seeded in 6-well plates (3 × 10^5^ cells/well) to 70–80% of the confluence.

The cells were resuspended in a new Petri dish and incubated overnight before treatment with high concentrations of glucose and drugs. To induce neuroinflammation, cells were exposed to high-glucose concentrations (25 mM), selected as described previously [27]. Microglial BV-2 cells were stimulated for 2, 8, 24, 48, and 72 h in RPMI with 3% FBS with D-glucose at 5.5 mM glucose, as a physiological blood glucose level (Normal Glucose, NG), and 25 mM glucose, to mimic conditions of hyperglycemia (High Glucose, HG). The effect produced by HG was compared with high mannitol concentration (5.5 mM glucose plus 19.5 mM mannitol, HM), used as an osmotic control treatment.

Authenticated human SH-SY5Y neuroblastoma cell line (A.T.C.C., Manassas, VA, USA; 10–20 passages) has been used to evaluate HG-mediated neurotoxicity. SH-SY5Y were cultured in a 1:1 combination of DMEM/F12 Ham’s nutrients medium (Merck, Darmstadt, Germany), under the same conditions as described above for BV-2 cells.

### 2.2. Plant Cell Culture and Phytocomplex Preparation

The stabilized and highly selected cell line specified on the synthesis of rosmarinic acid (RA) was derived from dissected young *Melissa officinalis* L. leaves, as described [28]. Briefly, the *M. officinalis* cell line was cultured to produce plant cell biomass in a bioreactor. At the end of the cell line growth in a bioreactor (10 days at 25 °C and in the dark), *M. officinalis* cell suspension was filtered through a 50 µm mesh filter, and the liquid culture medium was discarded. The cell biomass was washed with double the volume of saline solution (0.9% *w*/*v* NaCl in sterile water) and treated with 1% (*w*/*w*) of citric acid, after which it was homogenized using an ultraturrax at 15,000 rpm for 20 min. The homogenized cell biomass was dried to obtain a standardized phytocomplex (MO) with a high content of RA. Drying was performed using a Mini Spray Dryer (BUCHI-B290).

### 2.3. Phytocomplex Analysis and Characterization

MO was extracted and subjected to ^1^H NMR and UPLC-DAD analysis, performed by the methods described [29]. In addition, a qualitative-untargeted study using high-resolution mass spectrometry interfaced with UHPLC was performed. UHPLC-UV-ESI-MS is composed of a Q-Exactive Plus mass spectrometer (ThermoFisher Scientific, Waltham, MS, USA) equipped with an electrospray source (ESI) and coupled to a UHPLC Ultimate 3000SD (ThermoFisher Scientific, Waltham, MS, USA). The identification of individual peaks was performed using specific software that compares the signals obtained by mass spectrometry (full mass spectrum and MS/MS spectrum) with those present in the database of metabolites of the *Melissa officinalis*.

### 2.4. Drug Administration

HG-exposed BV-2 cells were treated with MO at 10 μg/mL or RA at 0.4 μg/mL for 48 h and compared to NG- and HG-treated cells. MO was dissolved in serum-free RPMI. The stock solution was then diluted in complete RPMI. RA (Merck, Darmstadt, Germany) was solubilized in cell culture medium at a concentration of 1 mg/mL, filtered (Filter syringe 0.2 m, 30 mm, Biosigma, Venice, Italy), and then diluted in the medium to obtain final concentrations of 0.4 µg/mL.

### 2.5. Cell Counting and Morphology Analysis

Cell morphological analysis was performed by experimenters blind to the cell culture conditions. After seeding in 6-well plates and stimulating BV-2 cells, images of each treatment were captured with an inverted light microscope (DM IL LED Fluo, Leica Microsystems) at the different timelines (2, 8, 24, 48, and 72 h of incubation). For each sowing were taken 3 photos per well. A total cell count was performed for each photo using Fiji ImageJ 2 14.0. By the ratio of the number of activated to the total number of cells, the percentage of BV-2 present in a proinflammatory state was obtained. In addition, cell length and soma area were measured, still using ImageJ. Cells were counted per mm^2^ microscopic area in at least 10 randomly selected fields. The experiments were conducted in three independent experimental sets. The average diameter and length of cellular processes, along with the soma surface area (average measurements from 120 individual cells), were evaluated. Cells were then grouped into three different categories: (a) small (<200 μm^2^), (b) mid-sized (200–400 μm^2^), and (c) large (>400 μm^2^) cells. For each experimental condition, a minimum of 50 cells was used for analysis.

### 2.6. SRB Cell Viability Assay

Sulforhodamine-B (SRB) assay has been used to detect cell viability. BV-2 cells were seeded in 96-well plates (2 × 10^4^ cells/well) and the various treatments (NG, HG, MD, AR) were performed. Forty-eight hours after treatment, cell media was removed; 100 μL/well of Hank Hanks’ Balanced Salt Solution (HBSS, Merck, Milan, Italy) was added, followed by 25 μL/well of 50% trichloroacetic acid (TCA) (Merck) and left to incubate at 4 °C for 1 h. Then five double-distilled water washes were carried out, after which the plate was left to dry, upside down, overnight at room temperature. The next day, staining was carried out; 25 μL/well of a solution of SRB 4 mg/mL in 1% acetic acid was added and allowed to incubate for 30 min at room temperature. Five washes were carried out using 200 μL/well of 1% acetic acid. Finally, 200 μL/well of a TRIS HCl solution at pH = 10 was added for five minutes after which the absorbance was read on a spectrophotometer at a wavelength of 570 nm. The treatments were performed in 6 replicates in 3 independent experiments, and cell viability was calculated by normalizing the values to the mean of the control.

### 2.7. MTT Assay

BV-2 cells have been stimulated with NG, HG, and HM for 48 h in RPMI with 3% FBS as previously described. Then, SH-SY5Y were sown in 96-well plates (2 × 10^4^ cells/well) and after stimulated with NG, HG, and HM-conditioned BV-2 media for 48 h. Finally, the cell viability test with MTT (3-(4,5-dimethylthiazol-2-yl)-2,5-diphenyl-2*H*-tetrazolium bromide) was performed. MTT is a colorimetric assay that exploits the conversion of a tetrazonium salt (yellow) to insoluble formazan crystals (violet) by succinate dehydrogenase. 20 µL/well of a 4 mg/mL solution of MTT (Merck, Milan, Italy) solubilized in DMSO was added and incubated for about 1 h at 37 °C and 5% CO_2_. Then everything is aspirated; 100 μL of pure DMSO is added to each well. 90 μL are taken, placed in a 96-well plate, and absorbance is read at 540 nm with a spectrophotometer; cell viability was calculated by normalizing to the NG group. Treatments were performed in 3 replicates in three independent experiments.

### 2.8. Protein Lysate from Cells

BV-2 were seeded in 6-well plates (3 × 10^5^ cell/well) and after treatment proteins from cells were extracted by RIPA buffer (50 mM Tris-HCl pH 7.4, 150 mM NaCl 1% sodium deoxycholate, 1% Triton X-100, 2 mM PMSF) (Merck, Milan, Italy) and the insoluble pellet was separated by centrifugation (12,000× *g* for 30 min, 4 °C). The total protein concentration in the supernatant was measured using the Bradford colorimetric method (Merck, Milan, Italy).

### 2.9. Western Blot

Protein samples (20–40 μg of protein/sample) were separated by 4–20% precast polyacrylamide gel electrophoresis (SDS-PAGE) and then blotted onto Midi Nitrocellulose membranes using a Trans-Blot Turbo Transfer Starter System (Biorad Laboratories, Milan, Italy). Membranes were blocked with PBST containing 5% non-fat dry milk for 60 min, then incubated overnight at 4 °C with primary antibodies: p-ERK (1:1000), IL-6 (1:500) (Santa Cruz Biotechnology, Dallas, TX, USA), and CD11b/ITGAM 1:1000 (Cell Signaling Technology, Danvers, MA, USA). The next day, membranes were washed three times with PBST and incubated for 2 h with HRP-conjugated secondary antibodies, then detected by a chemiluminescence detection system (Life Technologies, Monza, Italy). Signal intensity for each blot (pixels/mm^2^) was measured using ImageJ 2.14 (NIH). The signal intensity was normalized to that of total protein stained by Ponceau S. and protein expression was normalized to the mean of the control group (NG). Treatments were achieved in three independent experiments (n = 3).

### 2.10. Immunofluorescence Staining

BV2 cells are seeded in 24-well plates (1 × 10^5^ cells/well) until 70–80% confluence is reached. Forty-eight hours after treatments (NG, HG, MD, AR), BV2 cells were fixed with 4% PFA for 30 min at RT. Three washes with double-distilled water are performed after, which is added to a solution of 0.1% PBS-tryton (a cell membrane permeabilizer) for up to five minutes and then blocked with BSA 5% in PBST. The primary antibodies against iNOS (Cell Signaling, Danvers, MA, USA) and nuclear factor kB (NF-kB, Santa Cruz Biotechnology) were diluted 1:100 with BSA 1% in PBST overnight at +4 °C. Then, the secondary antibodies labeled with Alexa Fluor^®^ 488 AffiniPure Donkey Anti-Rabbit IgG (H + L) (Jackson ImmunoResearch Labs, West Grove, PA Cat# 711-546-152, RRID: AB_2340619) and Alexa Fluor^®^ 594 AffiniPure Goat Anti-Mouse IgG (H + L) (Jackson ImmunoResearch Labs Cat# 115-585-003) were applied for 1 h. Slides were mounted with 1 μg/mL of DAPI (nuclear marker) containing mounting medium (90% glycerol + 10% NaHCO_3_). Images were acquired using an OLYMPUS BX63F fluorescence microscope connected to a PC with an image acquisition card. The treatments were carried out in three independent experiments (n = 3), and the fluorescence intensity was calculated by normalizing the values to the mean of the control group (NG).

### 2.11. DPPH Radical Scavenging Assay

The radical scavenging activity of RA was determined spectrophotometrically by the 1,1-diphenyl-2-picryl-hydrazyl (DPPH) assay. RA was solved in methanol, and 100 µL of solution, at concentrations ranging from 1 to 100 µg/mL, were mixed with 100 μL of a DPPH solution in methanol (0.04 mg/mL). After 30 min of incubation at room temperature in the dark, the absorbance was measured at 517 nm. Each concentration was tested in triplicate. Ascorbic acid was used as a reference drug. Methanol served as a blank, and DPPH in methanol without RA served as a positive control. Background interferences from solvents were deducted from the activities of RA before calculating radical scavenging capacity as follows:

Radical scavenging activity (%) = [(Abs control − Abs sample)/Abs control] × 100

Abs stands for absorbance. Controls contained all the reaction reagents except RA or ascorbic acid.

### 2.12. Statistical Analysis

Results obtained are expressed as mean ± s.e.m. (standard error mean). Statistical analysis was performed using GraphPad Prism 10.0 (GraphPad Software Inc., San Diego, CA, USA) by means of an analysis of variance ANOVA, followed by the Tukey post hoc test. The values with *p*-values of *p* < 0.05 were considered significant.

## 3. Results

### 3.1. ^1^H NMR and UPLC-DAD Analysis of Melissa officinalis L. Phytocomplex from Plant Cell Cultures

**Figure 1:**
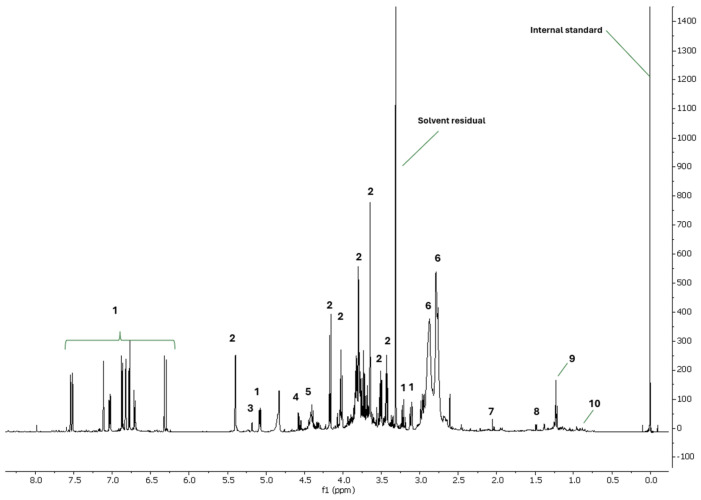
^1^H NMR profile of MO. The regions of δ 4.94−4.78, δ 3.34−3.30, 0.20−0.20 were excluded from the analysis because of the residual signals of solvents and standard. 1 = rosmarinic acid, 2 = sucrose, 3 = α-glucose, 4 = β-glucose, 5 = unknown sugar, 6 = citrate, 7 = acetate, 8 = alanine, 9 = ethanol, 10 = valine.

As previously described [29], the ^1^H NMR profiling of MO extract revealed the presence of both primary and secondary metabolites in MO. Specifically, sugars, amino acids, and organic acids were identified, including α-glucose, β-glucose, sucrose, alanine, valine, citrate, and acetate. Additionally, the aromatic region of the spectrum exhibited prominent signals ascribable to RA (Figure 1).

**Figure 2:**
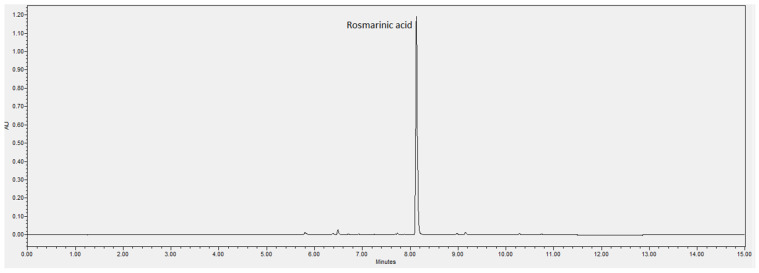
UPLC-DAD profile of the MO extract. The main peak at a retention time of 8.1 min corresponds to rosmarinic acid.

UPLC-DAD analysis was performed to estimate the total polyphenol content and the specific content of RA in the MO. Total polyphenols, expressed as equivalent of RA, were quantified by comparing peak areas measured at 330 nm wavelength to those of the calibration curve of the RA reference standard. The total polyphenol content, identified by its characteristic spectrum with λmax at 330 nm and expressed as RA equivalents, was 5.17 ± 0.1% *w*/*w*. The RA content, calculated by measuring the peak area at a retention time of 7.5, was 4.02 ± 0.1% *w*/*w*. The chromatographic profile of the MO is shown in Figure 2.

Untargeted analysis showed the presence of over 180 compounds. The most representative compound is RA, while the other metabolites are present in very low or trace amounts. The identified compounds present in the most relevant quantities are the following: malonyldidzin, quercitin 3-O-caffeyl-glucoside, theaflavin 3-O-gallate, leucosceptoside A, salvionolic acid B, isosalvionolic acid C, eugenin, ursolic acid, decaffeoylverbascoside, esculetin, 5-phenylvaleric acid, and oleanolic acid.

### 3.2. Time-Dependent BV2 Morphological Changes Induced by High-Glucose Exposure

BV2 cells were exposed to high glucose (HG; 25 mM) for 2, 8, 24, 48, or 72 h. As an osmotic control, cells were also incubated in an equal concentration of mannitol (HM; 25 mM) to rule out any potential effects due to hyperosmolarity. Normal glucose (NG; 5 mM) served as the control group.

**Figure 3:**
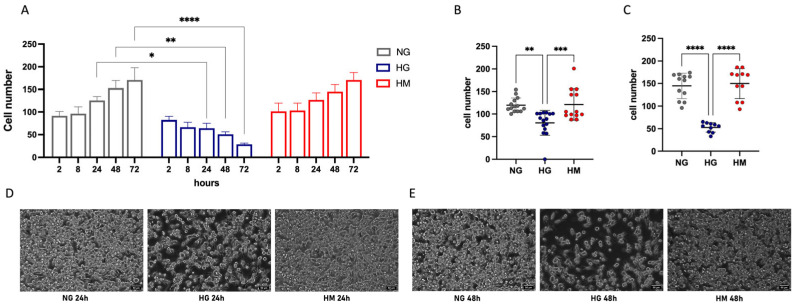
Time-dependent reduction in BV2 cell number by high glucose (HG) exposure. (**A**) BV2 cell number in cultures exposed to normal glucose (NG; 5.5 mM), high glucose (HG; 25 mM), or high mannitol (HM; 25 mM) concentrations at different time points (2, 8, 24, 48, and 72 h). Scatter plots of data obtained after 24 h (**B**) or 48 (**C**) hours of treatment. * *p* < 0.05, ** *p* < 0.01, *** *p* < 0.001, **** *p* < 0.0001. Representative images of HG-exposed cells after 24 h (**D**) and 48 h (**E**). Scale bar: 50 µm.

Firstly, we observed the effect of HG on the cell number. HG-exposed cells exhibited a progressive reduction of cell number over time compared to cells exposed to NG, which showed a progressive increase in cell number. HG-induced reduction of the cell number reached statistical significance at 24 h (125.4 ± 6.5 vs. 64.3 ± 10.5), 48 h (152.5 ± 20.0 vs. 50.8 ± 5.5), and 72 h (170.5 ± 27.0 vs. 28.3 ± 2.9). No significant changes were observed in the HM-exposed cells compared to the NG group (Figure 3A–C).

**Figure 4:**
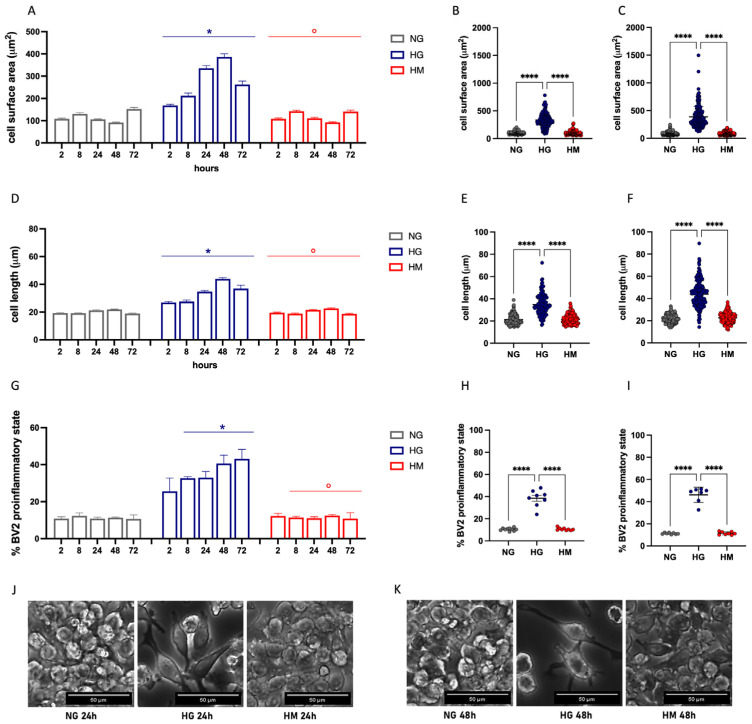
Morphological analysis of BV2 cells exposed to high glucose (HG). (**A**) Soma cell surface area of BV2 cells exposed to normal glucose (NG; 5.5 mM), high glucose (HG; 25 mM), or high mannitol (HM; 25 mM) concentrations at different time points (2, 8, 24, 48, and 72 h). * *p* < 0.05 vs. NG; ° *p* < 0.05 versus HG. Scatter plots of data obtained after 24 h (**B**) or 48 (**C**) hours of treatment. **** *p* < 0.0001. (**D**) BV2 cell length after exposure to NG, HG, and HM at different time points (2, 8, 24, 48, and 72 h). * *p* < 0.05 vs. NG; ° *p* < 0.05 versus HG. Scatter plots of data obtained after 24 h (**E**) or 48 (**F**) hours of treatment. **** *p* < 0.0001. (**G**) Increase in the percentage of cells in the proinflammatory state after stimulation with HG at different time points (2, 8, 24, 48, and 72 h) in comparison with NG and HM. * *p* < 0.05 vs. NG; ° *p* < 0.05 versus HG. Scatter plots of data obtained after 24 h (**H**) or 48 (**I**) hours of treatment. **** *p* < 0.0001. Representative images of HG-exposed cells after 24 h (**J**) and 48 h (**K**). Scale bar: 50 µm.

When BV2 cells were exposed to NG or HG, gross morphological differences in the cells between the two groups were observed. To systematically describe these differences, we conducted morphometric analyses of NG- and HG-exposed cells. Cells stimulated with HG displayed a time-dependent pro-inflammatory phenotype. BV2 cells can adopt two major morphological phenotypes: a short, rounded morphology and a stretched, elongated morphology. Unstimulated BV2 cells predominantly exhibited the round phenotype, but under HG exposure, the number of elongated cells significantly increased. After just 2 h of incubation, cells began to change morphology, adopting a prolonged, bipolar-like shape. Over time, these cells turned into a multipolar or prolonged shape with hypertrophied cell bodies and ramified or stellate morphology observed at 48 and 72 h. Detailed morphological characterization revealed that HG exposure progressively increased cell surface area (Figure 4A) and Feret’s diameter (Figure 4D), peaking at 24 h (Figure 4B: 3.16-fold to NG; Figure 4E: 1.64-fold to NG) and 48 h (Figure 4C: 4.19-fold to NG; Figure 4F: 2.0-fold to NG). The BV2 microglial cell lineage exhibits a broad heterogeneity, and some untreated control cells may already present a pro-inflammatory morphology. We evaluated the percentage of cells in a pro-inflammatory state, finding that about 10% of resting cells exhibited this phenotype, which increased dramatically after HG exposure to approximately 40–50% on average at 48 and 72 h (Figure 4G–I). The HM treatment did not alter any of the morphological parameters investigated.

Morphological analysis showed that the proinflammatory state peaked at 48 and 72 h after HG exposure. Due to the excessive reduction of cell number observed after 72 h, the following results refer to the evaluation of the efficacy of treatments on cells after 48 h exposure to HG.

### 3.3. Effect of MO and RA on Cell Viability

**Figure 5:**
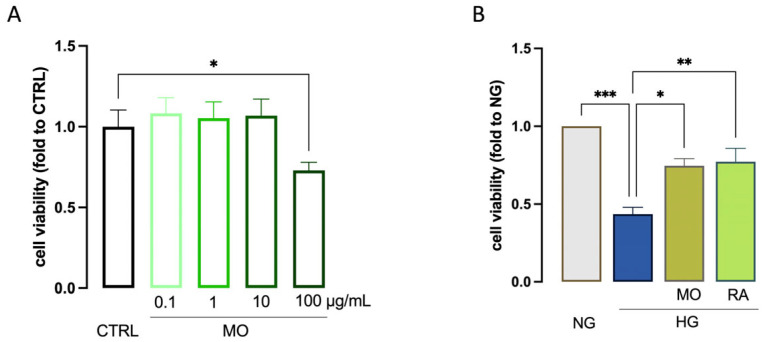
Effect of MO on cell viability. (**A**) Lack of alteration of cell viability by MO (0.1–100 µg/mL) in unstimulated BV2 cells. (**B**) MO (10 µg/mL) and RA (0.4 µg/mL) attenuation of HG-induced reduction of cell viability. * *p* < 0.05, ** *p* < 0.01*** *p* < 0.001.

No significant change in cell viability was observed after treatment with *M. officinalis* (MO) phytocomplex for 24 h up to the concentration of 100 µg/mL (Figure 5A). Thus, the effects produced by treatment were observed on stimulated cells. After exposure to HG, MO 10 µg/mL restored the significant reduction of cell viability induced by hyperglycemia (0.43 ± 0.04 vs. 0.71 ± 0.07). RA, administered at 0.4 µg/mL, which represents the amount found in the active concentration of MO, produced a comparable effect (0.43 ± 0.04 vs. 0.77 ± 0.08; Figure 5B). Thus, the concentration of MO 10 µg/mL was used for all experimentations.

### 3.4. MO and RA Reduced Proinflammatory Morphology Induced by High-Glucose Exposure

**Figure 6:**
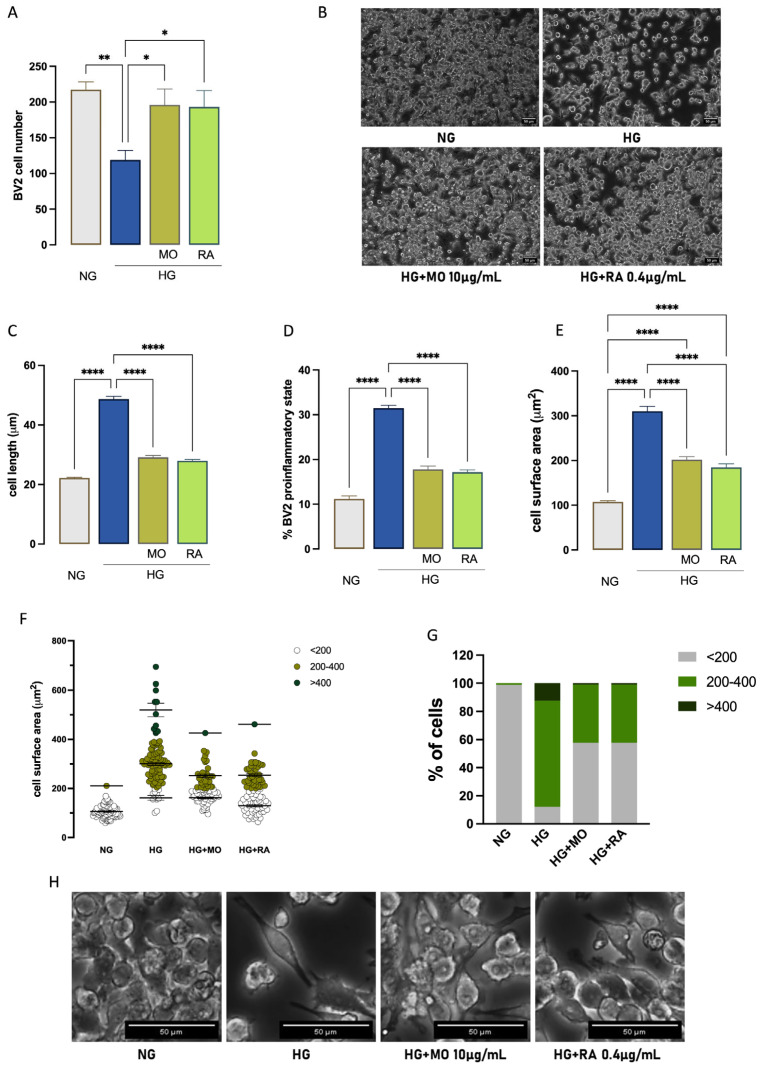
Attenuation by MO and RA of HG–induced proinflammatory morphological phenotype in BV2 cells. MO (10 µg/mL) and RA (0.4 µg/mL) attenuation of HG–induced cell number reduction (quantitative analysis (**A**); representative images (**B**)), cell length (**C**), percentage of cells in the proinflammatory state (**D**), and cell surface area (**E**). (**F**) Redistribution of HG–exposed BV2 cell subpopulations by cell surface area after 48 h of stimulation in the presence or absence of MO and RA. Cells were classified as either small (<200 μm^2^), mid-sized (200–400 μm^2^), or large (>400 μm^2^). (**G**) Effect of MO and CH on HG–induced variation of the percentage of distribution of cells by size. * *p* < 0.05, ** *p* <0.01, **** *p* < 0.0001. (**H**) Representative images of HG-exposed cells. Scale bar: 50 µm.

MO (10 µg/mL) and RA (0.4 µg/mL) counteracted the reduction in the cell number induced by HG exposure by restoring control levels (MO = 0.9, RA = 0.89 fold to NG; Figure 6A). Treatments also drastically reduced the cell diameter (HG = 2.2, MO = 1.3, RA = 1.26 fold to NG; Figure 6B), the number of cells in the proinflammatory state (HG = 2.8, MO = 1.5, RA = 1.5 fold to NG; Figure 6C), and the cell surface area (HG = 3.1, MO = 1.9, RA = 1.9 fold to NG; Figure 6D). The significant heterogeneity in cell area values across treatment groups led us to perform a distribution analysis to determine whether each treatment produced a distinct pattern of cell size distribution. We further analyzed the redistribution of cell subpopulations by surface area in each treatment group, identifying three major subgroups: small (<200 μm^2^) or medium-sized (200–400 μm^2^) and large (>400 μm^2^) cells (Figure 6E,F). In the unstimulated BV2 group, the majority of cells were small (approximately 98%). Under HG stimulation, the number of small-sized cells drastically decreased (12%), while there was a shift toward medium-sized and large cells (76% and 12%, respectively). Both treatments reduced the number of large and medium-sized cells and increased the number of small-sized cells (Figure 6E,F), indicating an anti-inflammatory effect.

MO and RA showed comparable efficacy.

### 3.5. Effect of MO and RA on Neuroinflammation Induced by High-Glucose Exposure

**Figure 7:**
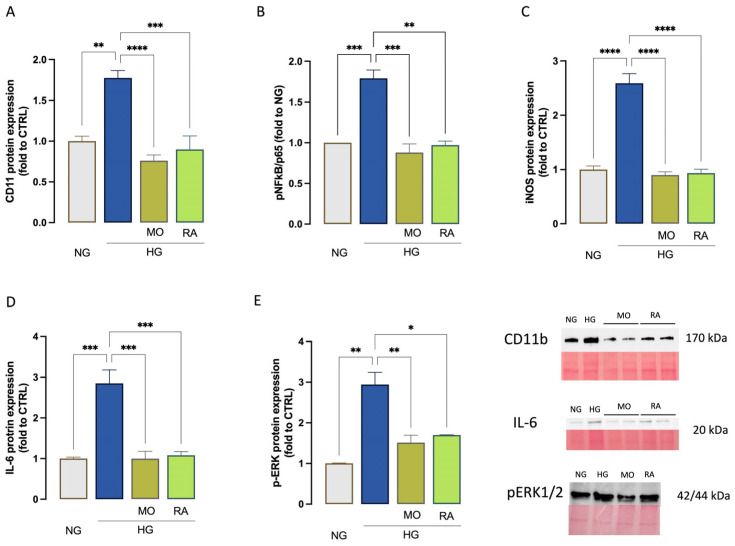
MO and RA inhibition of microglia activation and neuroinflammation. (**A**) Increase in CD11b expression by HG exposure and attenuation by MO (10 µg/mL) ND RA 0.4 µg/mL). Inhibition of HG-induced p65 overphosphorylation (**B**), induction of iNOS expression (**C**), IL-6 expression (**D**), and ERK1/2 (**E**) increased phosphorylation by MO and RA. * *p* < 0.05, ** *p* < 0.01, *** *p* < 0.001, **** *p* < 0.0001. Representative blots for western blotting analysis of CD11b, IL-6, and pERK1/2 are reported.

We first examined the effects of HG on the expression of CD11b, a marker of activated microglia. HG-exposed cells showed a robust overexpression of CD11b (1.8-fold to NG) that was downregulated by both treatments up to basal levels (0.8- and 0.9-fold to NG; Figure 7A). Then, the HG-induced phosphorylation of the p65 component, which is involved in the NF-κB canonical pathway [30], was investigated. Stimulated cells showed a dramatic increase in the p65 levels (1.8-fold to NG) that was completely abolished by MO and RA treatments (0.88- and 0.97-fold to NG; Figure 7B). We then examined the effects of HG on the expression of iNOS, a marker of nitro-oxidative stress that represents an inflammatory mediator whose expression in mammalian cells is predominantly governed by the NF-κB [31], thus commonly used also as a proinflammatory microglia marker. An overexpression of iNOS was detected in HG-exposed cells compared to NG-exposed cells (2.6-fold to NG). Pretreatment with both MO and RA downregulated the iNOS levels to baseline levels (0.9- and 0.93-fold to NG; Figure 7C). To confirm the potential anti-neuroinflammatory activity of investigated treatments, the levels of IL-6 were monitored. HG-exposed cells showed a robust increase in the IL-6 protein levels (2.8-fold to NG), completely restored by treatments (0.99- and 1.08-fold to NG; Figure 7D). Finally, the effect on MAPK levels was investigated. pERK1/2 levels raised in HG stimulated cells (2.9-fold to NG; Figure 7E). The levels of both MAPK were significantly reduced by MO and by RA (1.5- and 1.7-fold to NG; Figure 7E).

### 3.6. MO and RA Protect SH-SY5Y Cells from Neurotoxicity Induced by HG-Exposed BV-2 Microglial Cells

**Figure 8:**
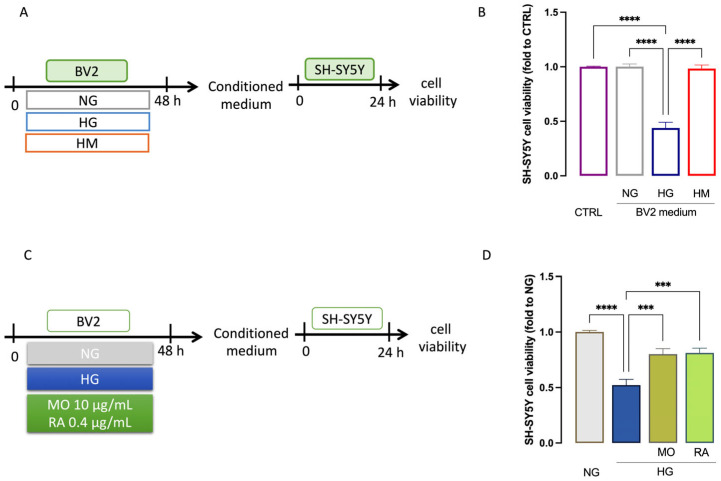
Neuroprotective effect of MO and RA on SH-SY5Y. (**A**) Schematic representation of experimental protocol and (**B**) reduction of cell viability in SH-SY5Y cells exposed to conditioned medium from HG-stimulated BV2 cells compared to NG- and HM-stimulated. CTRL cells were not exposed to the BV2 medium. (**C**) Schematic representation of experimental protocol and (**D**) protection by MO and RA treatment from neurotoxicity in SH-SY5Y cells induced by conditioned medium from HG-exposed BV2 cells. *** *p* < 0.001, **** *p* < 0.0001.

Neurons have a high metabolic rate, and neurobasal media containing 25 mM glucose meets these metabolic requirements. Optimal neuronal survival is observed with 25–30 mM basal glucose, while high glucose conditions contain at least 45 mM glucose [32]. Indeed, the glucose concentrations ranging from 20 to 25 mM represent the amount of glucose usually contained in the medium for neuronal cell culture, devoid of any effect on cell viability [33,34]. However, neurons can be exposed to microglia-released neuroinflammatory mediators with potential neurotoxic activity. To investigate whether MO and its main constituent RA protected neurons from the toxic effects of activated microglia, a conditioned medium for BV2 cells treated with HG alone or pretreated with MO or RA was collected and then co-cultured with SH-SY5Y cells to evaluate a potential neuroprotective activity. Exposure to conditioned medium from HG-treated BV2 cell cultures decreased the viability of SH-SY5Y cells compared to control cells that were not exposed to BV2 medium (0.43-fold). Conversely, medium from NG-exposed and HM-exposed BV2 cells did not produce any significant variation in cell viability (Figure 8A). Pretreatment with MO or RA for 24 h attenuated the neurotoxicity induced by HG (0.8 and 0.81 fold to NG; Figure 8B).

### 3.7. Antioxidant Activity of RA

**Figure 9:**
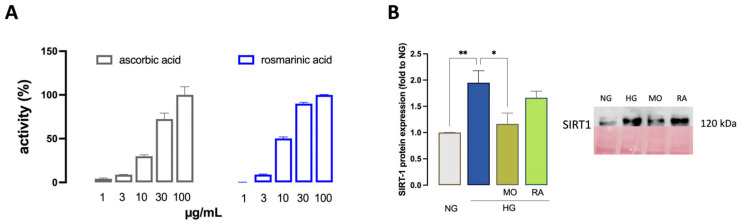
Antioxidant activity of MO and RA. (**A**) Anti-radical scavenger activity of rosmarinic acid (RA) (1–100 μg/mL) in the DPPH test. Ascorbic acid was used as a reference drug. (**B**) Reduction of the overexpression of SIRT1 by MO of HG-exposed BV2 cells. * *p* < 0.05, ** *p* < 0.01.

RA demonstrated an antiradical scavenger activity (1–100 µg/mL) comparable in intensity to that of ascorbic acid, which was used as the reference compound (Figure 9A). The antioxidant efficacy of RA and MO was also evaluated in living cells. BV2 cells exposed to HG showed an increased expression of SIRT1 protein (1.81-fold to NG), which was reduced to baseline levels by MO. RA produced a partial effect, suggesting that, although RA represents about 80% of the polyphenolic fraction of the phytocomplex, the antioxidant activity of MO is enhanced by other constituents present in MO (Figure 9B).

## 4. Discussion

Hyperglycemia, by activating microglia and resident immune cells in the brain, plays a key pathological role in promoting neuroinflammation [12,13], a phenomenon strongly correlated with the most common complications and comorbidities in diabetic patients [10]. In the present study, we characterized the proinflammatory phenotype of microglia under prolonged high glucose stimulation and examined the effects of an *M. officinalis* L. phytocomplex (MO) enriched in rosmarinic acid (RA) in dampening microglial neuroinflammation and promoting neuroprotection.

Microglia are highly dynamic cells in the brain that, to fulfill their functions, can exist in a variety of functional states or context-dependent phenotypes, thanks to their highly dynamic morphology and molecular profile [35]. These different microglial states reflect the cell’s response to specific stimuli and can be defined by a set of parameters, including cell morphology, molecular markers, and inflammatory mediators [35]. Morphological changes in microglial cells have been extensively described, with different phenotypes associated with resting microglia, which perform homeostatic functions, and proinflammatory microglia, which appear following exposure to a proinflammatory stimulus [36].

Glucose plays a crucial role in maintaining the metabolism of immune cells, and any alteration in blood glucose levels can impact microglial function. Several studies report that, upon HG stimulation, microglia undergo a morphological shift toward a proinflammatory phenotype [37,38,39,40]. Therefore, we first conducted a time-dependent morphological analysis of BV2 microglial cells under HG stimulation. Phenotypic changes in BV2 cells progressively occurred in response to prolonged HG exposure compared to cells exposed to NG (Figure 4). Pretreatment with MO counteracted these morphological changes and restored the microglial homeostatic phenotype (Figure 6). Previous studies have demonstrated the antidiabetic properties of *M. officinalis* extracts in both preclinical [41,42] and clinical [22,43] settings. By protecting against the development of neuroinflammation, MO also holds promise as an intervention for diabetes-associated neurological comorbidities. This hypothesis is further supported by a recent clinical study reporting an improvement in diabetes-associated depression and anxiety following treatment with a hydroalcoholic extract of *M. officinalis*.

MO is enriched in RA, a polyphenol that is one of the main constituents of *M. officinalis* associated with the plant’s key pharmacological functions [44,45]. Indeed, various biological activities have been attributed to this molecule, which is believed to underlie the well-documented antioxidant and anti-inflammatory properties of RA. Among its potential therapeutic effects, recent studies have highlighted some antidiabetic properties of RA, including its ability to improve insulin sensitivity and glucose uptake in animal models, as well as to restore blood glucose levels [23]. Due to its remarkable antioxidant properties, RA could also offer protection against diabetic nephropathy [46]. These studies suggest that the biological activities of RA mirror the effects observed for *M. officinalis* extracts. This hypothesis is further supported by our study, which demonstrated that RA exhibits efficacy comparable to that of MO (Figure 6). These findings suggest that the activity of the *M. officinalis* phytocomplex in restoring resting microglia morphology under hyperglycemic conditions is likely related to its RA content.

Although morphological changes provide relevant insights into the microglial phenotype, morphological analysis should not be equated with function [35], and it is important to correlate cell morphology with the analysis of proinflammatory mediators. Despite the heterogeneity in glucose concentration, exposure time, and microglial cell type in the studies published so far, there is a general consensus that HG exposure triggers the overexpression of markers for proinflammatory microglia, including ionized calcium-binding adapter molecule 1 (Iba1) [47,48] and CD11b [40,49]. Consistent with this, we observed a robust overexpression of CD11b in HG-treated cells compared to the normal glucose (NG) group, which was reversed by both MO and RA treatment (Figure 7A). NF-κB is widely considered the key driver of microglial activation. To investigate the molecular pathways activated by HG, we first examined the effect on NF-κB activation. A pronounced increase in the phosphorylation of the cytosolic NF-κB p65 subunit was observed, in agreement with previous studies [40]. NF-κB, in turn, governs the transcription and expression of numerous proinflammatory molecules. Among these, inducible nitric oxide synthase (iNOS), a marker of nitro-oxidative stress, plays a central role in regulating metabolism under stress conditions and inflammation [50]. iNOS is also deeply involved in promoting the microglial proinflammatory state [51], and its expression is predominantly regulated by NF-κB [31]. Consistent with this, we observed overexpression of iNOS in HG-treated cells, suggesting the activation of the NF-κB/iNOS proinflammatory pathway, which was completely restored by MO and RA treatment (Figure 7B,C).

HG treatment activates mitogen-activated protein kinase (MAPK) cascades, including the p38 MAPK pathway, and appears to activate a p38/NF-κB signaling pathway [52]. Additionally, HG promotes ERK1/2 phosphorylation [48], and the ERK1/2 inhibitor U0126 abrogated NF-κB activation in HG-treated BV2 cells [53], indicating the activation of a microglial ERK/NF-κB pathway by hyperglycemia. In our experimental conditions, we observed over-phosphorylation of ERK1/2 after HG exposure. MO was able to restore basal levels of MAPK proteins, and RA was equally effective in modulating pERK1/2 levels (Figure 7E), suggesting a prominent role for the ERK/NF-κB pathway in its mechanism of action.

In addition, HG exposure in microglia upregulates pro-inflammatory mediators, such as TNF-α [40,54], IL-6 [55,56], and IL-1β [37,57]. A robust overexpression of IL-6 was induced by HG exposure, further confirming the promotion of a neuroinflammatory response, which was completely abolished by both MO and RA with comparable efficacy (Figure 7D). Under conditions of chronic microglial stimulation, the accumulation of pro-inflammatory mediators becomes harmful to neighboring neurons, causing further microglial activation and leading to a vicious cycle [58]. Overexpression of pro-inflammatory mediators has been implicated in the development of neuroinflammation-related neuronal excitotoxicity [59] and neurodegenerative diseases [60], conditions that have been associated with diabetes [61]. Therefore, attenuating microglia-mediated neuroinflammation is a critical step in preventing neuronal injury and neurodegeneration. In this study, we demonstrated that exposure of neuronal cells to the HG-stimulated BV2-conditioned medium significantly reduced neuronal cell viability, an effect that was restored by pretreatment with MO or RA (Figure 6). Thus, dampening HG-induced microglia-mediated neuroinflammation using Melissa-based preparations may offer a potential therapeutic strategy for controlling hyperglycemia-associated neuroinflammation and neurodegeneration and, hopefully, for managing diabetes-associated neurological comorbidities [21].

Hyperglycemia exposes cells to high glucose concentrations and elevated osmotic pressure. Studies have shown that human fibroblasts and endothelial cells exposed to the high osmotic pressure associated with hyperglycemia exhibit reduced cell proliferation [62]. Similarly, microglia are highly sensitive to elevated osmotic pressure [63]. Therefore, the high osmotic pressure that accompanies acute hyperglycemia may impair microglial function, causing them to lose their homeostatic role in the brain. To exclude the possibility that the morphological and functional activation of microglia observed after HG exposure is due to a hyperosmotic mechanism, we treated cells with high mannitol (HM). The absence of any toxic effects in the HM-treated cells suggests that microglial activation is primarily related to a neuroinflammatory response.

LPS-stimulated BV2 cells are a widely used model of neuroinflammation. In this study, we used HG-stimulated BV2 cells as a model of hyperglycemia-induced neuroinflammation to test potential therapies aimed at counteracting neurological comorbidities in diabetic patients. Despite the undeniable limitations of using an immortalized cell system to approximate primary cell behavior in vitro, BV-2 cells have been shown to closely mimic the inflammatory response of primary microglia in vitro [64]. Furthermore, the response of BV-2 cells to proinflammatory stimuli shows substantial overlap with the response of primary cultured microglia and microglia in vivo [65].

## 5. Conclusions

High environmental glucose levels promote microglial activation, leading to a neuroinflammatory phenotype. BV2 cells exposed to HG exhibited morphological changes and activation of the NF-κB/iNOS pathway, as evidenced by increased phosphorylation of p65 and elevated iNOS expression. HG also activated the ERK1/2 signaling pathways and increased IL-6 protein expression. This neuroinflammatory response was prevented by MO. Clinical studies have shown that hydroalcoholic extracts of the aerial parts of *M. officinalis* can improve glycemic control, hyperlipidemia, and hypertension in diabetic patients. The present results suggest that MO, by regulating microglia activation induced by elevated glucose levels, has the potential for managing hyperglycemia-associated neurological complications. Moreover, using an MO phytocomplex obtained through plant cell culture could help address the variability in the chemical composition of herbal products, ensuring consistency in the composition of key constituents and reproducibility of clinical effects. This technique could also yield a phytocomplex with a higher content of RA, the active constituent primarily responsible for the therapeutic effects of MO.

## Data Availability

The data presented in this study are available on request from the corresponding author.
